# Comparison of characteristics of bimanual coordinated movements in older adults with frailty, pre-frailty, and robust health

**DOI:** 10.3389/fragi.2025.1519129

**Published:** 2025-02-27

**Authors:** Shoya Fujikawa, Shin Murata, Akio Goda, Shun Sawai, Ryosuke Yamamoto, Yusuke Shizuka, Takayuki Maru, Kotaro Nakagawa, Hideki Nakano

**Affiliations:** ^1^ Graduate School of Health Sciences, Kyoto Tachibana University, Kyoto, Japan; ^2^ Department of Rehabilitation, Kyoto Kuno Hospital, Kyoto, Japan; ^3^ Department of Physical Therapy, Faculty of Health Sciences, Kyoto Tachibana University, Kyoto, Japan; ^4^ Department of Physical Therapy, Faculty of Health and Medical Sciences, Hokuriku University, Kanazawa, Japan; ^5^ Department of Rehabilitation, Tesseikai Neurosurgical Hospital, Shijonawate, Japan; ^6^ Department of Rehabilitation, Junshinkai Kobe Hospital, Kobe, Japan; ^7^ Department of Rehabilitation, Nagashima Neurosurgery Rehabilitation Clinic, Osaka, Japan

**Keywords:** bimanual coordination, finger-tapping, older adults, frailty, pre-frailty, robust health

## Abstract

**Introduction:**

Despite the growing concern regarding a potential increase in the number of older adults with frailty owing to an aging global population, the characteristics of bimanual coordination in such older adults remain unclear. This study aimed to compare bimanual coordinated movements among community-dwelling older adults with frailty, pre-frailty, and robust health and identify the specific characteristics of these movements in older adults with frailty.

**Methods:**

Participants were categorized into frail, pre-frail, and robust groups based on Kihon Checklist scores. They performed bimanual coordination tasks in-phase (tapping the thumb and index finger together as fast as possible) and anti-phase (alternating the movement between the left and right fingers), and the task parameters were compared among the groups.

**Results:**

The total travel distance during the anti-phase task in the frail group was significantly shorter than that in the robust group. However, all three groups showed lower finger dexterity during the anti-phase task than in the in-phase task and the left hand than in the right hand.

**Conclusion:**

Older adults with frailty exhibit less movement during bimanual coordination tasks than robust older adults, suggesting that such tasks may be useful tools for assessing frailty.

## 1 Introduction

The percentage of older adults in the population is increasing annually worldwide. According to the World Health Organization, between 2020 and 2050, the population of individuals aged ≥60 years is estimated to double to 2.1 billion, and the population of those aged ≥80 years is projected to triple to 426 million (World Health Organization, 2022). The rapid aging of the global population has driven interest in improving the understanding of healthy aging and identifying assessment methods for it (Beard et al., 2016; Behr et al., 2023). Healthy aging is a complex multidimensional concept that encompasses biological, functional, lifestyle, and psychosocial factors (Behr et al., 2023). Additionally, achieving healthy aging requires early interventions to prevent significant declines in physical and cognitive functions (Silva et al., 2023). The number of older adults with frailty is also expected to increase as the older adult population grows. Frailty is defined as “a medical syndrome caused by multiple factors and triggers, characterized by a decline in muscle strength and endurance, a decrease in physiological function, and an increased vulnerability to needing care or facing death” (Morley et al., 2013). However, physical function in individuals with frailty has been reported to improve with appropriate interventions (de Labra et al., 2015). Furthermore, the prevention of frailty has been identified as a key future project in public health (Liotta et al., 2018) and holds significant social importance. Therefore, establishing a method for assessing frailty is crucial for maintaining the health of older adults.

In daily life, hands are the most frequently used body part (Lee and Jung, 2015), and healthy older adults have been found to engage in activities involving both hands more frequently than those involving only one hand (Kilbreath and Heard, 2005). Upper limb function in humans has been shown to change with age. Ingram et al. compared upper limb muscle strength, positional and superficial sensations, one-handed dexterity, bimanual coordination, muscle power stability, and functional performance in healthy participants aged 20–95 years (Ingram et al., 2019). Their results showed that the participant’s performance on all the parameters decreased with age, and the decline in bimanual coordination was particularly significant. Additionally, studies have reported that bimanual movements exhibit decreased accuracy, increased variability, and prolonged motor execution times with age (Kang et al., 2022). These results indicate that bimanual coordinated movements play an important role in the daily lives of older adults and that the coordination underlying these movements declines with age.

Although older adults with frailty have been reported to exhibit lower dexterity in one-handed movements than healthy older adults (Lammers et al., 2020; Schmidle et al., 2022), the characteristics of bimanual coordinated movements in older adults with frailty have not been clarified. Frailty in older adults has also been reported to result in less independence in activities of daily living than healthy older adults (Tornero-Quiñones et al., 2020), a higher risk of falling (Anders et al., 2007), and sarcopenia (Cruz-Jentoft and Sayer, 2019). In contrast, higher finger dexterity has been reported to be associated with better predictive postural control ability in stepping movements (Sun and Shea, 2016), and improved upper limb function has been reported to enhance gait ability and overall quality of life (Leblebici et al., 2024). Furthermore, sensory stimulation from the fingertips resulting from light contact has been shown to reduce ankle joint and body sway in the standing posture of older adults (Barela et al., 2018), suggesting that upper limb function, including finger function, can compensate for the decline in gait ability and standing balance. These findings indicate that bimanual coordination characteristics differ depending on the degree of frailty. Elucidating these differences could lead to the development of an assessment tool for the early detection of frailty. Therefore, this study aimed to compare the bimanual coordinated movements of community-dwelling older adults with frailty, pre-frailty, and robust health and determine the characteristics of bimanual coordinated movements in older adults with frailty. We hypothesized that the degree of frailty affects bimanual coordination, with bimanual coordination declining progressively from robust older adults to pre-frail older adults and then to frail older adults.

## 2 Methods

### 2.1 Participants

This cross-sectional study was conducted with 358 community-dwelling older adults who participated in physical fitness assessment sessions held in two cities in September 2023. The exclusion criteria for participants were: (i) age < 65 years; (ii) Mini-Mental State Examination (MMSE) scores < 24, based on previous studies (Mitchell, 2009; Ideno et al., 2012; Lin et al., 2014; Jin et al., 2019); (iii) presence of hand dexterity impairments due to musculoskeletal or central nervous system diseases; (iv) left-handedness; (v) inability to undergo measurements; and (vi) a maximum distance amplitude ≥ 300 mm in the bimanual coordination task (Enokizono et al., 2020). After applying these exclusion criteria, the remaining 312 participants were included in the analysis (Supplementary Figure S1).

### 2.2 Ethics declarations

This study was conducted in accordance with the principles of the Declaration of Helsinki and was approved by the Research Ethics Committee of Kyoto Tachibana University (Approval number 24–30). Informed consent was obtained from all the participants in the study.

### 2.3 Measures

First, we assessed the frailty of the participants using the Kihon Checklist (KCL). The KCL is a questionnaire developed in Japan to identify older adults at high risk of needing care in the near future (Arai and Satake, 2015; Satake et al., 2017). In recent years, the KCL has been widely used as a tool for assessing frailty. It has shown high sensitivity when validated against the Cardiovascular Health Study criteria and is regarded as the gold standard for frailty assessment (Satake et al., 2016). Accordingly, the KCL is recommended as a validated tool in international clinical guidelines for frailty assessment (Dent et al., 2017; Sentandreu-Mañó et al., 2021). The KCL is a self-administered questionnaire with “yes/no” responses that consists of 25 questions covering seven domains: activities of daily living, physical function, nutritional status, oral function, social withdrawal, cognitive function, and depressive mood. In the KCL, higher scores indicate a greater risk of needing care in daily life. In this study, participants with scores of 0–3, 4–7, and ≥8 were categorized into robust, pre-frail, and frail groups, respectively (Satake et al., 2016).

Next, all participants performed a bimanual coordination task. Participants sat on chairs with backrests and placed their forearms on a platform. During each task, the forearms were positioned in neutral rotation with the third, fourth, and fifth fingers slightly flexed, and the participants underwent measurements with their eyes closed (Supplementary Figure S2). The bimanual coordination task consisted of two tasks: the in-phase task, in which tapping movements of the thumb and index finger were performed simultaneously as quickly as possible with both hands, and the anti-phase task, in which tapping movements alternated between the left and right hands (Supplementary Figure S3) (Sano et al., 2011; Sugioka et al., 2020). The measurement process began with the in-phase task. Participants performed a 15-s practice session before the measurement, followed by a 15-s measurement for each task. Adequate rest was provided between tasks to prevent participants from becoming fatigued. All measurements were completed in approximately 5 min. We instructed all participants to “perform as fast as possible and maintain the same rhythm” during the bimanual coordination tasks. During the practice session, we confirmed that participants had no communication problems, fully understood the task content, and were able to perform the task accurately, as explained by the experimenter.

Finger movements during bimanual coordination tasks were measured using a magnetic sensor finger-tapping device (UB-2, Maxell Ltd. Tokyo, Japan) (Sugioka et al., 2020). This device comprises a magnetic induction coil, a sensing coil, and a circuit unit (Kandori et al., 2004). The sensors are attached to the participant’s thumb and index finger using sensor attachment bands, and voltage is induced between them based on electromagnetic induction. Since the induced voltage has a nonlinear relationship with the distance between the coils, the distance between the fingertips where the sensors are attached can be estimated from the voltage (Shima et al., 2008). Therefore, the magnetic sensor finger-tapping device provides highly reproducible and reliable measurements across periods, devices, and examiners (Sano et al., 2011). During the bimanual coordination task, the participants were instructed to open their fingers to a width of 40 mm to minimize amplitude variations across participants (Suzumura et al., 2021; Sugioka et al., 2022). The parameters of the bimanual coordination task (distance, tap interval, and phase difference) were obtained from the recorded data (Table 1) (Sano et al., 2011). Four parameters of “Distance” were used to evaluate the distance and movement amplitude of the thumb and index finger during the task; four parameters of “Tap interval” were used to evaluate the average speed of movement and variability of tapping; and one parameter of “Phase difference” was used to evaluate the timing discrepancy of tapping between the hands.

**TABLE 1:** Characteristics of the bimanual coordinated task.

	Parameter	Description	Assessment
Distance	Total travel distance (mm)	The sum of the distances moved by the thumb and index finger. The overall amount of movement.	Higher values indicate higher finger dexterity.
	Ave of local max distance (mm)	Average amplitude of the distance waveform.	Values closer to 40 mm indicate higher finger dexterity.
	SD of local max distance (mm)	Variation in the amplitude of the distance waveform.	Lower values indicate higher finger dexterity.
	Slope of approximate line of local max points (mm/s)	The slope is a linear regression of the relationship between the maximum point of each tap and time. As the tap amplitude decreases due to fatigue, the slope increases in the negative direction. When there is no effect of fatigue, the slope is 0.	Lower values indicate higher finger dexterity.
Tap interval	Number of taps (taps)	Number of taps during the measurement time.	Higher values indicate higher finger dexterity.
	Ave of tap intervals (s)	Average in time difference between two consecutive taps.	Lower values indicate higher finger dexterity.
	Frequency of taps (Hz)	Inverse to the mean of the tap interval.	Higher values indicate higher finger dexterity.
	SD of inter-tap interval (s)	Variations in time difference between two consecutive taps.	Lower values indicate higher finger dexterity.
Phase difference	SD of phase difference (degree)	Assuming the interval between one tap is 360°, the time lag between the left and right hands is expressed as an angle. This parameter is the variation of its value.	Lower values indicate higher finger dexterity.

Ave, average; Max, maximum; SD, standard deviation.

### 2.4 Statistical analysis

Participants were categorized into frail, pre-frail, and robust groups based on the KCL results. First, a chi-square test was conducted to compare the male/female ratios among the groups. Participants’ age, height, weight, and MMSE and KCL scores were compared between the groups using one-way analysis of variance (ANOVA). Next, three-way ANOVA with a mixed design was conducted to compare the total travel distance, average of local maximum distance, standard deviation (SD) of local maximum distance, slope of the approximate line of local maximum points, number of taps, average of tap intervals, frequency of taps, and SD of inter-tap interval during the bimanual coordination task, considering hand (left, right), task (in-phase task, anti-phase task), and group (frail, pre-frail, robust) as factors. Additionally, a two-way ANOVA with a mixed design was used to compare the SD of the phase difference between left- and right-hand tapping, considering task (in-phase task, anti-phase task) and group (frail, pre-frail, robust) as factors. Bonferroni *post hoc* tests were performed for parameters showing significant interactions or main effects in all ANOVAs. Finally, Pearson correlation analysis was conducted to examine the relationship between the bimanual coordination tasks and the MMSE, assessing whether participants’ cognitive function influenced bimanual coordination. Statistical analyses were performed using SPSS version 29.0 (IBM, Armonk, NY, United States), with the significance level set at 5%.

## 3 Results

### 3.1 Characteristics of the participants

Based on the KCL assessment of frailty, we categorized the participants into three groups: frail (47 participants; 8 males, 39 females; aged 69–90 years), pre-frail (136 participants; 27 males, 109 females; aged 65–91 years), and robust (129 participants; 33 males, 96 females; aged 65–93 years). The results of the chi-square test showed no significant differences among male/female ratios in each group (χ^*2*^ = 2.10, *p* = 0.37). One-way ANOVA revealed no significant intergroup differences in age, height, weight, or MMSE score (*p* > 0.05) but showed significant differences in the KCL score (*p* < 0.05). Post-hoc tests showed that the KCL scores in the pre-frail and frail groups were significantly higher than that of the robust group, and the score in the frail group was significantly higher than that in the pre-frail group (*p* < 0.05; Table 2).

**TABLE 2:** Characteristics of the participants.

	Frail (n = 47)	Pre-frail (n = 136)	Robust (n = 129)	F	Post-hoc test
Age (years)	78.38 (5.73)	77.94 (6.25)	76.85 (6.04)	1.57	
Height (cm)	152.09 (8.77)	152.92 (7.60)	154.07 (8.32)	1.26	
Body weight (kg)	50.41 (8.27)	52.48 (9.87)	53.70 (9.46)	2.13	
MMSE (score)	27.74 (1.99)	28.35 (1.76)	28.46 (1.85)	2.68	
KCL (score)	9.47 (1.70)	5.10 (1.07)	1.91 (0.99)	772.14*	Robust < Pre-frail < Frail

MMSE, mini-mental state examination; KCL, kihon checklist; *p < 0.05.

### 3.2 Results of three-way ANOVA with hand, task, and group as factors

The three-way ANOVA results showed no significant interactions among the three factors (hand × task × group) for the total travel distance, average of local maximum distance, SD of local maximum distance, slope of the approximate line of local maximum points, number of taps, average of tap intervals, frequency of taps, and SD of the inter-tap interval (*p* > 0.05). Additionally, no significant hand × group and task × group interactions were observed. Conversely, the slope of the approximate line of local maximum points and the SD of the inter-tap interval showed significant hand × task interactions (*p* < 0.05). Post-hoc test results indicated that in the in-phase task, the slope of the approximate line of local maximum points was significantly higher in the right hand than in the left hand (*p* < 0.05). Additionally, the slope of the approximate line of the local maximum points in the right hand was significantly higher in the in-phase task than in the anti-phase task (*p* < 0.05). The SD of the inter-tap interval was significantly higher in the left hand than in the right hand in both the in-phase and anti-phase tasks (*p* < 0.05). In addition, the SD of the inter-tap interval was significantly higher in the anti-phase task than in the in-phase task for both the left and right hands (*p* < 0.05).

The total travel distance showed a significant main effect of the group factor (*p* < 0.05). Post-hoc test results indicated that the total travel distance was significantly longer in the robust group compared to the frail group (*p* < 0.05) (Supplementary Figure S4). The total travel distance, average of the local maximum distance, SD of the local maximum distance, slope of the approximate line of local maximum points, number of taps, average tap intervals, frequency of taps, and SD of the inter-tap interval had significant main effects of the task factor (*p* < 0.05). The *post hoc* test results showed that the total travel distance, number of taps, and frequency of taps showed significant main effects of the task factor (*p* < 0.05). The frequency of taps was significantly higher in the in-phase task than in the anti-phase task (*p* < 0.05). The average of the local maximum distance, SD of the local maximum distance, and average tap intervals were significantly higher in the anti-phase task than in the in-phase task (*p* < 0.05). The total travel distance, SD of the local maximum distance, number of taps, average tap intervals, frequency of taps, and SD of the inter-tap interval showed significant main effects of the hand factor (*p* < 0.05). According to the *post hoc* tests, the total travel distance, number of taps, and frequency of taps were significantly higher for the right hand than for the left hand (*p* < 0.05). In contrast, the SD of the local maximum distance and average tap intervals were significantly higher for the left hand than for the right hand (*p* < 0.05; Table 3).

**TABLE 3:** Results of three-way ANOVA with hand, task, and group as factors.

	Task	Hand	Frail (n = 47)	Pre-frail (n = 136)	Robust (n = 129)	IE	IE	IE	IE	ME	ME	ME	Post-hoc test		
						Hand × group	Task × group	Hand × task	Hand × group × task	Hand	Task	Group			
						F	F	F	F	F	F	F	Hand	Task	Group
Total traveling distance (mm)	IP	L	3,857.11	4,125.05	4,599.64	2.48	0.46	0.71	1.02	6.99*	105.45*	3.78*	L < R^*b*^	AP < IP^*b*^	Frail < Robust^*b*^
			(1,491.55)	(1,550.39)	(1,658.75)										
		R	4,145.72	4,339.03	4,565.60										
			(1,301.58)	(1,506.82)	(1,555.99)										
	AP	L	3,342.03	3,475.23	3,820.00										
			(1,221.67)	(1,099.30)	(1,269.37)										
		R	3,478.85	3,637.13	3,842.62										
			(1,345.38)	(1,218.38)	(1,259.59)										
Ave of local max distance (mm)	IP	L	44.59	48.02	48.49	1.24	1.20	0.52	0.55	0.61	296.09*	0.37		IP < AP^*b*^	
			(14.92)	(17.47)	(16.69)										
		R	46.64	49.35	47.59										
			(10.78)	(16.78)	(15.84)										
	AP	L	61.17	60.83	63.15										
			(18.47)	(16.07)	(17.13)										
		R	61.34	62.08	62.54										
			(16.40)	(16.84)	(16.27)										
SD of local max distance (mm)	IP	L	6.07	6.83	6.72	0.23	0.84	0.39	1.49	33.41*	50.52*	2.14	R < L^*b*^	IP < AP^*b*^	
			(1.71)	(2.40)	(2.22)										
		R	5.64	5.66	5.64										
			(1.62)	(2.06)	(2.09)										
	AP	L	7.16	7.97	7.78										
			(3.48)	(3.46)	(2.96)										
		R	6.27	7.35	7.03										
			(3.11)	(3.34)	(2.80)										
Slope of approximate line of local max points (mm/s)	IP	L	−0.09	−0.08	−0.16	0.69	0.21	7.49*	0.78	3.25	4.33*	0.06	IP: L < R^*a*^	R: AP < IP^*a*^	
			(0.48)	(0.71)	(0.68)										
		R	0.03	0.06	0.05										
			(0.61)	(0.67)	(0.58)										
	AP	L	−0.05	−0.18	−0.13										
			(0.77)	(0.75)	(0.81)										
		R	−0.18	−0.11	−0.13										
			(0.55)	(0.75)	(0.72)										
Number of taps	IP	L	43.32	43.79	46.94	0.53	0.46	0.32	0.13	20.05*	590.79*	2.74	L < R^*b*^	AP < IP^*b*^	
			(12.57)	(14.58)	(13.32)										
		R	43.72	44.77	47.81										
			(12.67)	(15.54)	(14.11)										
	AP	L	26.64	27.69	29.60										
			(8.37)	(7.77)	(9.36)										
		R	27.13	28.40	30.13										
			(9.10)	(8.15)	(9.79)										
Ave of intervals (s)	IP	L	0.37	0.39	0.35	0.12	1.88	0.52	0.05	4.52*	339.70*	1.50	R < L^*b*^	IP < AP^*b*^	
			(0.12)	(0.17)	(0.13)										
		R	0.37	0.38	0.34										
			(0.12)	(0.17)	(0.13)										
	AP	L	0.61	0.57	0.56										
			(0.24)	(0.17)	(0.23)										
		R	0.60	0.56	0.55										
			(0.21)	(0.17)	(0.24)										
Frequency of taps (Hz)	IP	L	2.93	2.96	3.17	0.76	0.52	0.80	0.06	17.43*	583.26*	2.73	L < R^*b*^	AP < IP^*b*^	
			(0.84)	(0.97)	(0.89)										
		R	2.95	3.03	3.23										
			(0.84)	(1.04)	(0.95)										
	AP	L	1.83	1.90	2.02										
			(0.56)	(0.52)	(0.62)										
		R	1.84	1.94	2.05										
			(0.61)	(0.54)	(0.66)										
SD of inter-tapping interval (s)	IP	L	0.04	0.04	0.04	0.02	0.27	5.48*	0.53	36.49*	129.20*	0.03	IP: R < L^*a*^ AP: R < L^*a*^	L: IP < AP^*a*^ R: IP < AP^*a*^	
			(0.03)	(0.03)	(0.03)										
		R	0.03	0.04	0.04										
			(0.02)	(0.02)	(0.03)										
	AP	L	0.09	0.09	0.10										
			(0.07)	(0.07)	(0.08)										
		R	0.08	0.07	0.08										
			(0.08)	(0.05)	(0.08)										

Ave, average; Max, maximum; SD, standard deviation; IP, in-phase task; AP, anti-phase task; L, left; R, right, IE, interaction effect; ME, main effect; *a*, *post hoc* test of interaction effect; *b*, *post hoc* test of main effect; * *p* < 0.05.

### 3.3 Results of two-way ANOVA with task and group as factors

In the two-way ANOVA, the SD of the phase difference showed no significant interaction between the task and group factors nor a main effect of the group factor. However, a significant main effect of the task factor was observed (*p* < 0.05). The *post hoc* test results showed that the SD of the phase difference was significantly higher for the anti-phase task than for the in-phase task (*p* < 0.05; Table 4).

**TABLE 4:** Results of two-way ANOVA with task and group as factors.

	Task	Frail (n = 47)	Pre-frail (n = 136)	Robust (n = 129)	IE	ME	ME	Post-hoc test	
					Task × group	Task	Group		
					F	F	F	Task	Group
SD of phase difference (degree)	IP	26.46	27.99	29.68	0.12	36.99*	0.41	IP < AP^*b*^	
		(18.70)	(17.26)	(25.57)					
	AP	37.87	37.59	38.93					
		(24.32)	(21.15)	(18.86)					

SD, standard deviation; IP, in-phase task; AP, anti-phase task; L, left; R, right; IE, interaction effect; ME, main effect; *b*, *post hoc* test of main effect; * *p* < 0.05.

### 3.4 Results of correlation analysis between the bimanual coordination task and the MMSE

The results of the correlation analysis showed that the total traveling distance, the slope of the approximate line of local maximum points, number of taps, average tap intervals, frequency of taps, and SD of inter-tapping intervals in the in-phase and anti-phase tasks were significantly correlated with the MMSE; however, the correlations were very weak (*p* < 0.05; Supplementary Table S1).

## 4 Discussion

This study compared the characteristics of bimanual coordinated movements in community-dwelling older adults with frailty, pre-frailty, and robust health. The results showed that the total distance of the bimanual coordinated movements was shorter in the frail group than in the robust group. Additionally, regardless of the degree of frailty, finger dexterity during the bimanual coordination task was lower in the anti-phase task than in the in-phase task and lower in the left hand than in the right hand. These results suggest that older adults with and without frailty exhibit similar levels of bimanual coordination. However, the amount of movement in bimanual coordination tasks was lower in older adults with frailty than in robust older adults. Bimanual coordination tasks are simple, non-invasive, and can be performed without placing an excessive burden on older adults. These characteristics make bimanual coordination tasks suitable as an assessment method for older adults living in the community or those with limited mobility, enabling evaluations to be conducted at home or in caregiving settings. Based on these points, assessing the total traveling distance during bimanual coordination tasks holds potential as a screening method to identify frail older adults.

### 4.1 Relationship between bimanual coordination and frailty

In this study, older adults were categorized into pre-frail, frail, and robust groups and asked to perform a bimanual coordination task consisting of in-phase and anti-phase tasks. The results showed that the total travel distance was shorter in the frail group than in the robust group. The total travel distance is influenced by the velocity and number of movement taps (Tomita et al., 2020), which represent the amount of finger movement. In a study evaluating the relationship between frailty and finger movement control while performing unilateral movements using the dominant hand, agility, smoothness of movement, and strength were reported to be lower in older adults with frailty than in healthy older adults (Schmidle et al., 2022). Frailty is characterized by a decline in muscle strength and endurance, and reduced muscle strength results in slower movement speeds (Morley et al., 2013; Alcazar et al., 2019). Therefore, it is likely that the frail group performed bimanual coordination tasks more slowly than the robust group. Consequently, the total traveling distance in the frail group may have been shorter than that of the robust group in this study. Although the difference in the amount of movement between the frail and robust groups could also be attributed to reduced endurance in the frail group (Morley et al., 2013; Angulo et al., 2020), the slope of the approximate line of the local maximum points, which reflects the effect of fatigue based on the relationship between the maximum distance between two fingers per tap and time, showed no significant difference between the frail and robust groups in this study. Therefore, the reduced movement in the bimanual coordination task was likely owing to decreased muscle strength and slower movement speed rather than a decline in endurance.

### 4.2 Comparison of in-phase and anti-phase tasks

Our findings also showed that the total travel distance, number of taps, and frequency of taps were higher in the in-phase task than in the anti-phase task. The average of the local maximum distance, SD of the local maximum distance, average tap intervals, and SD of the phase difference were higher in the anti-phase task than in the in-phase task. The slope of the approximate line of the local maximum points is suspected to be influenced by fatigue because the distance between the two fingers becomes narrower over time if the slope has a negative value. In the present study, the slope of the approximate line of the local maximum points was negative for the anti-phase task and positive for the in-phase task for the right hand. Therefore, the anti-phase task may have been affected by fatigue. Additionally, the SD of the inter-tap interval showed that the rhythm of movement was more variable in the anti-phase task. The number of taps, average tap interval, and frequency of taps indicated that the anti-phase task involved fewer taps, a lower frequency, and longer periods than the in-phase task. The total travel distance and the average and SD of the local maximum distance revealed that the amount of movement was smaller in the anti-phase task than in the in-phase task and that the distance between two fingers per tap and its variation were larger in the anti-phase task than in the in-phase task. The SD of the phase difference showed that the anti-phase task had more timing deviations than the in-phase task for both tasks. Therefore, in this study, the performance of the anti-phase task was lower than that of the in-phase task for all parameters of finger dexterity. The anti-phase task requires specific muscle activity with continuous timing to maintain alternating bimanual movements, and this timing is asymmetric between the left and right hands (Tian et al., 2020). Additionally, maintaining attention is necessary to preserve the phase relationship between hands. For anti-phase tasks and cognitive function, research involving community-dwelling older adults with declining cognitive function has shown a correlation between tapping velocity in the anti-phase task and a decline in working memory and attention (Suzumura et al., 2021). Therefore, the anti-phase task, which requires independent alternating movements of both hands, is suggested to be more challenging than the in-phase task or unilateral motor tasks and is prone to differences in finger function (Sugioka et al., 2022). Therefore, similar to robust older adults, older adults with frailty in this study may have experienced higher difficulty in the anti-phase task than in the in-phase task and showed characteristics of reduced finger dexterity for each parameter.

### 4.3 Comparison of left and right hand in bimanual coordinated movement

Since the participants performed the same finger-tapping task with their left and right hands, we expected no significant differences between the parameters for each hand. However, the total travel distance was significantly longer with the right hand than with the left. Additionally, the SD of the local maximum distance was lower for the right hand than for the left hand. The right hand showed a higher number and frequency of taps as well as longer intervals than the left hand. Furthermore, the SD of the inter-tap interval was smaller for the right hand than for the left hand. If the thumb is repeatedly moved in a specific direction, the trained movement increases cortical excitability (Classen et al., 1998). Therefore, repetitive movements induce plastic reorganization in the primary motor cortex, and this phenomenon is called use-dependent plasticity (Mawase et al., 2017; Raffin and Siebner, 2019). This use-dependent plasticity has been found to inhibit motor errors and reduce motor planning time, even in complex daily activities (Spampinato and Celnik, 2021). The dominant hand is used more frequently than the non-dominant hand in daily life, and older adults are trained to use the dominant hand in their daily activities (Suzumura et al., 2016). These findings suggest that the primary motor cortex innervating the dominant hand enables spatially and temporally efficient movements through use-dependent plasticity (Shin et al., 2009). In the present study, the right hand may have had higher finger dexterity than the left hand for all parameters, regardless of other factors. Therefore, older adults with frailty, such as robust older adults, have higher finger dexterity during bimanual coordination tasks with their right hand than with their left hand.

### 4.4 Limitations

This study had a few limitations. First, the KCL consists of seven domains: activities of daily living, motor function, nutritional status, oral function, social withdrawal, cognitive function, and depressive mood. It provides a simple and multidimensional approach to evaluating frailty. However, this study did not clarify how bimanual coordination is related to the physical, social, and psychological aspects of frailty. This point requires further investigation. Second, in this study, the cutoff value for the MMSE was set at less than 24 points, which means the study may have also included older adults with mild cognitive impairment. In the future, it will be necessary to clarify the characteristics of bilateral coordination in older adults with mild cognitive impairment or cognitive frailty, which is a combination of mild cognitive impairment and physical frailty. Third, this study did not examine in detail whether the participants were able to perform the bimanual coordination task accurately. In the future, it will be necessary to examine the accuracy of the bimanual coordination task, including reliability and reproducibility. Fourth, this study investigated the characteristics of bimanual coordination only at the behavioral level and did not examine the neural mechanisms underlying bimanual coordination. Previous studies comparing unilateral movements across a wide range of ages, from children to healthy older adults, have demonstrated that immature or degenerated motor systems may maintain or improve performance by bilaterally mobilizing brain regions, as opposed to normal motor systems (Addamo et al., 2013; Fujiyama et al., 2016). Moreover, structural changes in the corpus callosum have been observed in older adults with frailty (Sugioka et al., 2022), suggesting that a decline in bimanual coordination may be attributed to alterations in interhemispheric interaction. Future studies should investigate interhemispheric interactions and functional and structural changes in the corpus callosum among frail older adults using transcranial magnetic stimulation or functional magnetic resonance imaging. Fifth, while the magnetic sensor-based finger-tapping device used in this study enables precise measurements, it is costly and may be difficult to implement in certain settings. This limitation, particularly in resource-constrained environments, may hinder the wider application of this method. It is important to explore and develop alternative, more cost-effective methods capable of measuring similar parameters to address this limitation. Such efforts could enhance the practicality and accessibility of frailty assessments across diverse environments.

## 5 Conclusion

This study characterized bimanual coordinated movements in older adults with frailty, pre-frailty, and robust health. Based on the bimanual coordination task, the total traveling distance was shorter in the frail group than in the robust group. Regardless of the severity of frailty, participants showed lower bimanual coordination in the anti-phase task than in the in-phase task, and finger dexterity during the bimanual coordination tasks was lower in the left hand than in the right hand. Therefore, while older adults with frailty exhibit bimanual coordination similar to that of robust older adults, the amount of movement in the bimanual coordination task by those with frailty is lower than that of robust older adults. The results of this study suggest that bimanual coordination tasks may be applicable as an assessment tool for frailty.

## Data Availability

The data supporting the findings of this study are available from the corresponding author upon reasonable request. The data are not publicly available because they contain information that could compromise the privacy of research participants.

## References

[B1] Addamo P. K. Farrow M. Bradshaw J. L. Moss S. Georgiou-Karistianis N. (2013). Characterizing the developmental profile of effort-induced motor overflow across a timed trial. Am. J. Psychol. 126, 227–234. 10.5406/amerjpsyc.126.2.0227 23858955

[B2] Alcazar J. Csapo R. Ara I. Alegre L. M. (2019). On the shape of the force-velocity relationship in skeletal muscles: the linear, the hyperbolic, and the double-hyperbolic. Front. Physiol. 10, 769. 10.3389/fphys.2019.00769 31275173 PMC6593051

[B3] Anders J. Dapp U. Laub S. von Renteln-Kruse W. (2007). Impact of fall risk and fear of falling on mobility of independently living senior citizens transitioning to frailty: screening results concerning fall prevention in the community. Z. Gerontol. Geriatr. 40, 255–267. 10.1007/s00391-007-0473-z 17701116

[B4] Angulo J. El Assar M. Álvarez-Bustos A. Rodríguez-Mañas L. (2020). Physical activity and exercise: strategies to manage frailty. Redox Biol. 35, 101513. 10.1016/j.redox.2020.101513 32234291 PMC7284931

[B5] Arai H. Satake S. (2015). English translation of the Kihon checklist. Geriatr. Gerontol. Int. 15, 518–519. 10.1111/ggi.12397 25828791

[B6] Barela A. M. F. Caporicci S. de Freitas P. B. Jeka J. J. Barela J. A. (2018). Light touch compensates peripheral somatosensory degradation in postural control of older adults. Hum. Mov. Sci. 60, 122–130. 10.1016/j.humov.2018.06.001 29883963

[B7] Beard J. R. Officer A. de Carvalho I. A. Sadana R. Pot A. M. Michel J.-P. (2016). The World report on ageing and health: a policy framework for healthy ageing. Lancet 387, 2145–2154. 10.1016/S0140-6736(15)00516-4 26520231 PMC4848186

[B8] Behr L. C. Simm A. Kluttig A. Grosskopf Großkopf A. (2023). 60 years of healthy aging: on definitions, biomarkers, scores and challenges. Ageing Res. Rev. 88, 101934. 10.1016/j.arr.2023.101934 37059401

[B9] Classen J. Liepert J. Wise S. P. Hallett M. Cohen L. G. (1998). Rapid plasticity of human cortical movement representation induced by practice. J. Neurophysiol. 79, 1117–1123. 10.1152/jn.1998.79.2.1117 9463469

[B10] Cruz-Jentoft A. J. Sayer A. A. (2019). Sarcopenia. Lancet 393, 2636–2646. 10.1016/S0140-6736(19)31138-9 31171417

[B11] de Labra C. Guimaraes-Pinheiro C. Maseda A. Lorenzo T. Millán-Calenti J. C. (2015). Effects of physical exercise interventions in frail older adults: a systematic review of randomized controlled trials. BMC Geriatr. 15, 154. 10.1186/s12877-015-0155-4 26626157 PMC4667405

[B12] Dent E. Lien C. Lim W. S. Wong W. C. Wong C. H. Ng T. P. (2017). The Asia-Pacific clinical practice guidelines for the management of frailty. J. Am. Med. Dir. Assoc. 18, 564–575. 10.1016/j.jamda.2017.04.018 28648901

[B13] Enokizono T. Ohto T. Tanaka M. Maruo K. Sano Y. Kandori A. (2020). Quantitative assessment of fine motor skills in children using magnetic sensors. Brain Dev. 42, 421–430. 10.1016/j.braindev.2020.03.004 32249080

[B14] Fujiyama H. Van Soom J. Rens G. Gooijers J. Leunissen I. Levin O. (2016). Age-related changes in frontal network structural and functional connectivity in relation to bimanual movement control. J. Neurosci. 36, 1808–1822. 10.1523/JNEUROSCI.3355-15.2016 26865607 PMC6602021

[B15] Ideno Y. Takayama M. Hayashi K. Takagi H. Sugai Y. (2012). Evaluation of a Japanese version of the mini-mental state examination in elderly persons. Geriatr. Gerontol. Int. 12, 310–316. 10.1111/j.1447-0594.2011.00772.x 22122408

[B16] Ingram L. A. Butler A. A. Walsh L. D. Brodie M. A. Lord S. R. Gandevia S. C. (2019). The upper limb physiological profile assessment: description, reliability, normative values and criterion validity. PLoS One 14, e0218553. 10.1371/journal.pone.0218553 31247034 PMC6597070

[B17] Jin Y. Seong J. Cho Y. Yoon B. (2019). Effects of aging on motor control strategies during bimanual isometric force control. Adapt. Behav. 27, 267–275. 10.1177/1059712319849896

[B18] Kandori A. Yokoe M. Sakoda S. Abe K. Miyashita T. Oe H. (2004). Quantitative magnetic detection of finger movements in patients with Parkinson’s disease. Neurosci. Res. 49, 253–260. 10.1016/j.neures.2004.03.004 15140567

[B19] Kang N. Ko D. K. Cauraugh J. H. (2022). Bimanual motor impairments in older adults: an updated systematic review and meta-analysis. EXCLI J. 21, 1068–1083. 10.17179/excli2022-5236 36381648 PMC9650695

[B20] Kilbreath S. L. Heard R. C. (2005). Frequency of hand use in healthy older persons. Aust. J. Physiother. 51, 119–122. 10.1016/s0004-9514(05)70040-4 15924514

[B21] Lammers F. Zacharias N. Borchers F. Mörgeli R. Spies C. D. Winterer G. (2020). Functional connectivity of the supplementary motor network is associated with Fried’s modified frailty score in older adults. J. Gerontol. A Biol. Sci. Med. Sci. 75, 2239–2248. 10.1093/gerona/glz297 31900470

[B22] Leblebici G. Tarakcı E. Kısa E. P. Akalan E. Kasapçopur Ö. (2024). The effects of improvement in upper extremity function on gait and balance in children with upper extremity affected. Gait Posture 110, 41–47. 10.1016/j.gaitpost.2024.02.017 38484646

[B23] Lee K.-S. Jung M.-C. (2015). Quantitative comparison of marker attachment methods for hand motion analysis. Int. J. Occup. Saf. Ergon. 21, 30–38. 10.1080/10803548.2015.1017960 26327260

[B24] Lin C.-H. Chou L.-W. Wei S.-H. Lieu F.-K. Chiang S.-L. Sung W.-H. (2014). Influence of aging on bimanual coordination control. Exp. Gerontol. 53, 40–47. 10.1016/j.exger.2014.02.005 24548774

[B25] Liotta G. Ussai S. Illario M. O’Caoimh R. Cano A. Holland C. (2018). Frailty as the future core business of public health: report of the activities of the A3 action group of the European Innovation Partnership on Active and Healthy Ageing (EIP on AHA). Int. J. Environ. Res. Public Health. 15, 2843. 10.3390/ijerph15122843 30551599 PMC6313423

[B26] Mawase F. Uehara S. Bastian A. J. Celnik P. (2017). Motor learning enhances use-dependent plasticity. J. Neurosci. 37, 2673–2685. 10.1523/jneurosci.3303-16.2017 28143961 PMC5354321

[B27] Mitchell A. J. (2009). A meta-analysis of the accuracy of the mini-mental state examination in the detection of dementia and mild cognitive impairment. J. Psychiatr. Res. 43, 411–431. 10.1016/j.jpsychires.2008.04.014 18579155

[B28] Morley J. E. Vellas B. van Kan G. A. Anker S. D. Bauer J. M. Bernabei R. (2013). Frailty consensus: a call to action. J. Am. Med. Dir. Assoc. 14, 392–397. 10.1016/j.jamda.2013.03.022 23764209 PMC4084863

[B29] Raffin E. Siebner H. R. (2019). Use-dependent plasticity in human primary motor hand area: synergistic interplay between training and immobilization. Cereb. Cortex 29, 356–371. 10.1093/cercor/bhy226 30364930 PMC6294416

[B30] Sano Y. Kandori A. Shima K. Tamura Y. Takagi H. Tsuji T. (2011). “Reliability of finger tapping test used in diagnosis of movement disorders,” in 2011 5th international conference on bioinformatics and biomedical engineering, Wuhan, China, 10–12 May, 2011 (IEEE), 1–4. 10.1109/icbbe.2011.5780409

[B31] Satake S. Senda K. Hong Y.-J. Miura H. Endo H. Sakurai T. (2016). Validity of the Kihon checklist for assessing frailty status. Geriatr. Gerontol. Int. 16, 709–715. 10.1111/ggi.12543 26171645

[B32] Satake S. Shimokata H. Senda K. Kondo I. Toba K. (2017). Validity of total Kihon checklist score for predicting the incidence of 3-year dependency and mortality in a community-dwelling older population. J. Am. Med. Dir. Assoc. 18, 552.e1–552.e6. 10.1016/j.jamda.2017.03.013 28479274

[B33] Schmidle S. Gulde P. Herdegen S. Böhme G.-E. Hermsdörfer J. (2022). Kinematic analysis of activities of daily living performance in frail elderly. BMC Geriatr. 22, 244. 10.1186/s12877-022-02902-1 35321645 PMC8943928

[B34] Sentandreu-Mañó T. Cezón-Serrano N. Cebrià I Iranzo M. A. Tortosa-Chuliá M. A. Tomás J. M. Salom Terrádez J. R. (2021). Kihon checklist to assess frailty in older adults: some evidence on the internal consistency and validity of the Spanish version. Geriatr. Gerontol. Int. 21, 262–267. 10.1111/ggi.14126 33393211

[B35] Shima K. Tsuji T. Kan E. Kandori A. Yokoe M. Sakoda S. (2008). Measurement and evaluation of finger tapping movements using magnetic sensors. Annu. Int. Conf. IEEE Eng. Med. Biol. Soc. 2008, 5628–5631. 10.1109/IEMBS.2008.4650490 19163993

[B36] Shin H.-W. Sohn Y. H. Hallett M. (2009). Hemispheric asymmetry of surround inhibition in the human motor system. Clin. Neurophysiol. 120, 816–819. 10.1016/j.clinph.2009.02.004 19299196

[B37] Silva N. Rajado A. T. Esteves F. Brito D. Apolónio J. Roberto V. P. (2023). Measuring healthy ageing: current and future tools. Biogerontology 24, 845–866. 10.1007/s10522-023-10041-2 37439885 PMC10615962

[B38] Spampinato D. Celnik P. (2021). Multiple motor learning processes in humans: defining their neurophysiological bases. Neuroscientist 27, 246–267. 10.1177/1073858420939552 32713291 PMC8151555

[B39] Sugioka J. Suzumura S. Kawahara Y. Osawa A. Maeda N. Ito M. (2020). Assessment of finger movement characteristics in dementia patients using a magnetic sensing finger-tap device. Jpn. J. Compr. Rehabil. Sci. 11, 91–97. 10.11336/jjcrs.11.91

[B40] Sugioka J. Suzumura S. Kuno K. Kizuka S. Sakurai H. Kanada Y. (2022). Relationship between finger movement characteristics and brain voxel-based morphometry. PLoS One 17, e0269351. 10.1371/journal.pone.0269351 36206254 PMC9543950

[B41] Sun R. Shea J. B. (2016). Probing attention prioritization during dual-task step initiation: a novel method. Exp. Brain Res. 234, 1047–1056. 10.1007/s00221-015-4534-z 26708519

[B42] Suzumura S. Kanada Y. Osawa A. Sugioka J. Maeda N. Nagahama T. (2021). Assessment of finger motor function that reflects the severity of cognitive function. Fujita. Med. J. 7, 122–129. 10.20407/fmj.2020-013 35111556 PMC8761821

[B43] Suzumura S. Osawa A. Nagahama T. Kondo I. Sano Y. Kandori A. (2016). Assessment of finger motor skills in individuals with mild cognitive impairment and patients with Alzheimer’s disease: relationship between finger-to-thumb tapping and cognitive function. Jpn. J. Compr. Rehabil. Sci. 7, 19–28. 10.11336/jjcrs.7.19

[B44] Tian Q. Williams O. A. Landman B. A. Resnick S. M. Ferrucci L. (2020). Microstructural neuroimaging of frailty in cognitively normal older adults. Front. Med. 7, 546344. 10.3389/fmed.2020.546344 PMC7645067 33195297

[B45] Tomita Y. Tanaka S. Takahashi S. Takeuchi N. (2020). Detecting cognitive decline in community-dwelling older adults using simple cognitive and motor performance tests. Geriatr. Gerontol. Int. 20, 212–217. 10.1111/ggi.13863 31917896

[B46] Tornero-Quiñones I. Sáez-Padilla J. Espina Díaz A. Abad Robles M. T. Sierra Robles Á. (2020). Functional ability, frailty and risk of falls in the elderly: relations with autonomy in daily living. Int. J. Environ. Res. Public Health. 17, 1006. 10.3390/ijerph17031006 32033397 PMC7037456

[B47] World Health Organization (2022). Ageing and health. Available at: https://www.who.int/news-room/fact-sheets/detail/ageing-and-health.

