# Supplementary material for: Comparison of characteristics of bimanual coordinated movements in older adults with frailty, pre-frailty, and robust health
Source: Front Aging. 2025 Feb 27;6:1519129. doi: 10.3389/fragi.2025.1519129 (PMC11903717; doi:10.3389/fragi.2025.1519129)
Supplement: Supplementary file 1 [file DataSheet1.docx]

Supplementary Material

# Supplementary Figures

**Supplementary Figure 1. Flowchart of participant selection.** Participants aged < 65 years, with Mini-Mental State Examination scores < 24, hand dexterity impairments due to musculoskeletal or central nervous disease, left-handedness, inability to undergo all measurements, or maximum distance amplitude ≥ 30 cm in the measured data of the bimanual coordination task were excluded. Finally, this study included 312 participants.


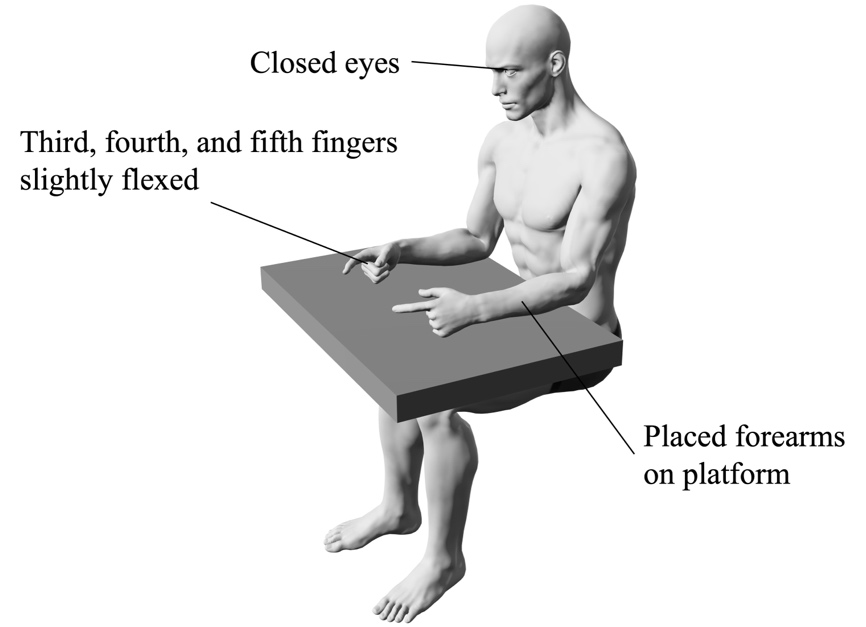


**Supplementary Figure 2. Measurement position in the bimanual coordination task.** Participants sat on chairs with backrests and placed their forearms on the platform. During each task, the forearms were positioned in neutral rotation, with the third, fourth, and fifth fingers slightly flexed, and participants underwent measurements with closed eyes.


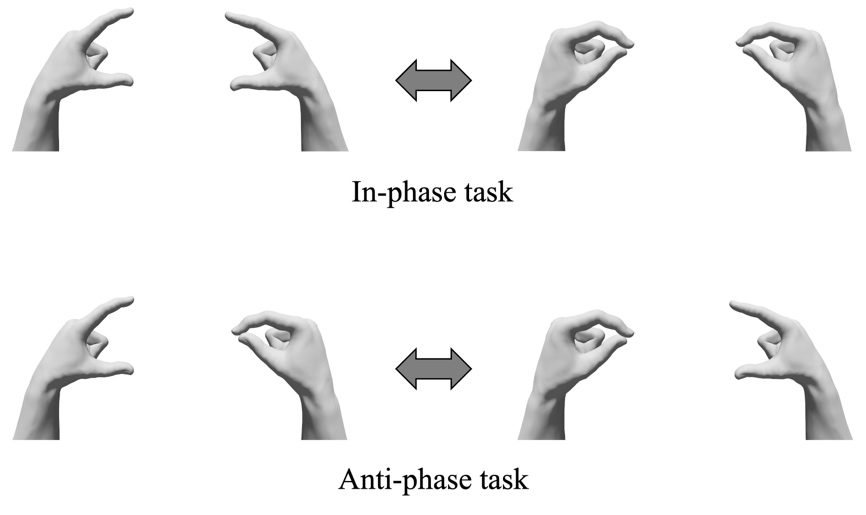


**Supplementary Figure 3. Bimanual coordination task.** (A) In-phase task: Participants performed tapping movements of the thumb and index finger simultaneously on both sides. (B) Anti-phase task: Participants performed tapping movements of the thumb and index finger alternately on both sides.

**Supplementary Figure 4. The results of the three-way ANOVA on the total traveling distance.** The total traveling distance showed a significant main effect on the group (*p* < 0.05). Post-hoc tests revealed that the robust group had a significantly longer total traveling distance than the frail group (^*^***p*** < 0.05). A significant main effect of the hand was found (p < 0.05), indicating that the total traveling distance was significantly longer for the right hand than for the left hand (^†^***p*** < 0.05). Furthermore, a significant main effect of the task was also observed (*p* < 0.05), with the total traveling distance being significantly longer during the in-phase task compared to the anti-phase task (^‡^***p*** < 0.05). IP, In-phase task; AP, Anti-phase task.

# Supplementary Tables

**Supplementary Table 1. Correlation analysis between the bimanual coordination task and the MMSE**

|  | Task | Total traveling distance (mm) | | Ave of local max distance (mm) | | SD of local max distance (mm) | | Slope of an approximate line of local max　points (mm/s) | | Number of taps | | Ave of intervals (s) | | Frequency of taps (Hz) | | SD of inter-tapping interval (s) | | SD of phase difference (degree) |
| --- | --- | --- | --- | --- | --- | --- | --- | --- | --- | --- | --- | --- | --- | --- | --- | --- | --- | --- |
|  |  | L | R | L | R | L | R | L | R | L | R | L | R | L | R | L | R |  |
| Coefficient | IP | 0.15^*^ | 0.11^*^ | 0.06 | 0.02 | -0.08 | -0.06 | 0.06 | 0.12^*^ | 0.16^*^ | 0.15^*^ | -0.14^*^ | -0.13^*^ | 0.15^*^ | 0.15^*^ | -0.11^*^ | -0.11^*^ | 0.06 |
|  | AP | 0.20^*^ | 0.16^*^ | -0.02 | -0.05 | -0.06 | -0.02 | 0.00 | 0.11^*^ | 0.24^*^ | 0.23^*^ | -0.21^*^ | -0.20^*^ | 0.25^*^ | 0.23^*^ | -0.19^*^ | -0.14^*^ | -0.11 |

IP, in-phase task; AP, anti-phase task; Ave, average; Max, maximum; SD, standard deviation; L, left; R, right, ^*^*p* < 0.05.
